# Diversity Analysis of Global White Clover (*Trifolium repens* L.) Germplasm Based on Agronomic and Photosynthetic Traits and SLAF-Seq Technology

**DOI:** 10.3390/ijms27114882

**Published:** 2026-05-28

**Authors:** Ruxue Sang, Maryam Noor, Guilan Feng, Mengli Han, Yuxi Feng, Peichun Mao, Xuebing Yan, Lin Meng

**Affiliations:** 1 Institute of Grassland, Flowers and Ecology, Beijing Academy of Agriculture and Forestry Sciences, No. 9 Shugguang Huayuan Middle Road, Haidian District, Beijing 100097, China; 2 College of Animal Science and Technology, Yangzhou University, Yangzhou 225009, China

**Keywords:** white clover (*Trifolium repens* L.), germplasm resources, SNP marker, SLAF sequencing, population structure

## Abstract

Based on SLAF-seq technology, 174 white clover accessions were analyzed using population structure and genetic evolution to develop SNP markers of all accessions. We obtained 2329.4 Mb reads of sequenced data, and the reads of the samples ranged from 4,701,984 to 31,540,232. The sequencing quality value (Q30) uniformly changed from 90.61% to 96.82%, with an average of 93.11%. The GC content of the samples changed from 38.96% to 43.98%, averaging 40.96%, with a control of 34.21%. A total of 320,417 SLAF tags were developed, with an average sequencing depth of 16.42×. There were 202,625 polymorphic SLAF tags, accounting for 63.24% of the total number of SLAF tags. Finally, 2,999,555 polymorphic SNPs were found, and 102,025 high-quality SNPs were selected for downstream analyses after filtering with minor allele frequency (MAF) > 0.05 and completeness > 0.5. Population structure analysis supported K = 2, indicating two major ancestral genetic backgrounds among the accessions. Phylogenetic analysis and principal component analysis further divided the accessions into three genetic subclusters, suggesting finer-scale genetic differentiation. In addition, one-way ANOVA and chi-squared tests revealed a significant association between genetic groups and geographic origin (χ^2^ = 25.78, df = 8, *p* = 0.0012; F = 3.489, *p* = 0.032), provided limited evidence for a possible association between genetic grouping and geographic origin. Compared with photosynthetic traits, agronomic traits showed a broader range of variations, with coefficient of variance values for agronomic traits ranging from 24.59% to 139.02% and for photosynthetic traits from 4.29% to 78.57%. This difference suggests that morphological traits were highly differentiated among the 174 accessions. The consistency between phenotypic clustering (based on agronomic traits) and molecular clustering (based on SNP data) suggests that our SNP dataset captures biologically meaningful genetic variation, providing a solid foundation for future genome-wide association studies (GWASs) and marker-assisted selection (MAS) in white clover.

## 1. Introduction

White clover (*Trifolium repens* L.) serves as a significant fodder legume in temperate-region pastures. This species is an allotetraploid (2*n* = 4x = 32) distinguished by its stoloniferous growth pattern [1]. White clover developed through multiple hybridization procedures in Mediterranean glacial refugia distinguished by fertile soils, adequate precipitation, and grazing animals, which facilitated its spread across Europe, western Asia, and into northern Africa [2,3]. White clover genotypes and ecotypes are primarily categorized on a leaf-size basis, e.g., small, intermediate, large and ladino types. Small-leaved genotypes are optimal for sheep grazing, medium-sized for rotational grazing, and large for ruminant grazing or silage preparation [4,5]. Globally, breeders are focusing on the contribution and persistency of white clover with grasses to mixed swards [6]. Pasture forage breeding has made important contributions to improving forage yield, persistence, nutritive value, and adaptation to diverse production environments, but further progress requires the effective exploitation of genetic variation preserved in germplasm resources [7]. A prior agronomic advantage of clover is its nitrogen fixation capability using atmospheric N through symbiosis with *Rhizobium*, a root nodule bacterium. It increases available nitrogen to grasslands by helping in soil fertility maintenance and increase in crop yield alongside companion grasses in grasslands [8,9]. Furthermore, *T*. *repens* itself acts as a rich source of nitrogen [10] by increasing the nutritive values of grass mixed with white clover forages compared to grass alone [11]. In addition, grasses mixed with clover attain comparable or higher productivity compared to monoculture grasses because of different functional characteristics and resources use complementarity. *T*. *repens* can fix almost 150–280 kg N ha^−1^ and is suitable for both grazing and fodder [12]. Grass mixed with clovers can increase aboveground biomass, crop dry matter, and organic matter compared to grasses alone [13,14]. White clover plus perennial ryegrass increases topsoil-lodging earthworms by improving soil structure, helping in root growth and deep penetration [13]. In addition, in summers it serves as an excellent food source for honeybees, bumblebees, and other bee species [12,15]. Clover and some other Fabaceae members produce flowers that contain long corolla tubes that repel hoverflies [16]. Because white clover is commonly grown in mixed-pasture systems, its genetic variation may influence not only individual plant performance but also plant–plant interactions, resource use, and ecological adaptation within grassland communities [17].

The main limitations of white clover growth are (i) competition with different grass species, (ii) pest attack, (iii) opposition from nitrogen source, (iv) humidity, (v) drought stress, (vi) and low temperature [18]. *T*. *repens* possesses an extensive range of genomic variation in leaf and root structure, nutrient absorption, and tolerance against pathogens and environmental stresses. This morphogenetic variation allows the cultivars to survive across varied geography and ecological regions [19]. In forage cultivars like white clover, agronomic traits including leaf size, leaf number, and internode length [20] and photosynthetic (physiological) indicators like photosynthetic capacity and stomatal conductance are vital both individually and synergistically for enhancing dry-matter production, fodder quality, and long-term survival (tolerance under stress) [21]. Agronomic traits are normally affected by climatic conditions, so their expressions vary among different environments. Therefore, without deep understanding the genetic base of these traits, it is difficult to assess how different cultivars can perform under fluctuating environmental conditions [22,23]. This oversight may lead to selection of cultivars that perform excellently in a particular environment, but are unsuitable for others, restraining the adaptation and resilience of new genotypes [24]. Relying only on agronomic traits may result in short-term improvement, but limits the prospects for long-term genomic gains [25]. The incorporation of genetic bases enables the discovery of alleles linked with desirable features, and hence aids in achieving smarter and more targeted breeding methodologies. This strategy can increase the cumulative genetic advancement of white clover across successive breeding cycles [26,27]. DNA-based approaches have become powerful tools for assessing genetic diversity in grassland plant species, providing more stable and genome-wide information than morphology-based evaluation alone [28]. Recently, high-throughput sequencing-based technologies have enabled high-density, precise genotyping and offer novel approaches for studying plant genetic traits. Among these, SLAF-seq (specific-locus amplified fragment sequencing) has emerged as an efficient method for large-scale SNP discovery. This technique has been successfully applied in various crops for purposes such as SNP marker development, construction of high-resolution genetic maps, and QTL mapping of agronomically important traits [29]. Moreover, SLAF-seq integrates careful in silico design, size-selective library creation, double-barcode multiplexing, and deep but targeted sequencing, facilitating reliable and scalable SNP detection and genotyping even in individuals without a reference genome. It is exclusively advantageous in plant breeding, QTL mapping, and genome-wide studies, even in non-model crops such as white clover [30,31]. SNP markers are more effective genetic markers than traditional molecular markers because they are the most abundant and stable form of genetic variation in most genomes to compare SNP markers with traditional marker systems, such as SSRs or AFLPs [32]. Currently, simplified genome sequencing technologies that have been reported include restriction enzyme site-associated DNA (RAD) sequencing [33], type IIB restriction endonuclease site-associated DNA (double-digest RAD-seq) sequencing [34], genotyping by sequencing (GBS) [35], and specific length-amplified fragment sequencing (SLAF-seq) techniques, which have one thing in common, i.e., reducing the complexity of genomic DNA by restriction endonucleases [36].

This study employed SLAF-seq technology to develop SNP molecular markers across 174 white clover germplasm accessions, achieving high genome-wide coverage. The primary objectives were: (i) assessment of phenotypic diversity of key agronomic and photosynthetic traits in a globally diverse white clover germplasm panel, (ii) to characterize the genome-wide genetic diversity and population structure using SLAF-seq-derived SNPs, (iii) and to discuss implications for white clover breeding and germplasm management.

## 2. Results and Analysis

### 2.1. Analysis of Variation in Morphological Indicators in the Germplasm of White Clover

Basic statistical analysis of nine morphological characteristics of 174 white clover germplasm results showed that there were great differences among different accessions, with obvious morphological diversity. The plant height of the tested materials ranged from 5.9 to 24.13 cm, with an average of 12.19 cm. The leaf length ranged from 1.25 to 4.94 cm, with an average of 1.83 cm, and leaf width varied from 0.98 to 6.9 cm, with an average of 1.79 cm. The leaf length measured was 1.25 to 4.94 cm, with a mean of 1.83 cm. The petiole length was from 5.45 to 22.68 cm, and averaged 10.96 cm. The diameter of the plants ranged from 0.75 to 3.21 cm, with an average of 1.48 cm. Overall, the coefficient of variation of the number of stolons was highest, reaching 139.02%, followed by stolon density, leaf area, and stolon length, with coefficients of variation of 116.58%, 113.36%, and 107.51%, respectively. The variation in leaf width was relatively small, 40.22%, while there was a slight difference in leaf length, with a CV of 24.59% (Table 1).

### 2.2. Principal Component Analysis of Morphological Traits of White Clover Germplasm

Principal component analysis was conducted on nine morphological traits to identify the major contributors to phenotypic variation among *Trifolium repens* genotypes worldwide. Three principal components with eigenvalues greater than 1.0 were extracted, and the cumulative contribution rate of the first three principal components reached 85.75%, indicating that these components explained most of the morphological variation among the tested germplasm materials.

Based on the overall contribution hierarchy across PC1–PC3, leaf area showed the highest contribution, accounting for 12.32%, followed by leaf width, petiole length, leaf length, and plant height, with contributions of 12.22%, 12.19%, 12.18%, and 12.16%, respectively. These five traits were mainly associated with PC1 and together accounted for 61.07% of the total contribution, suggesting that leaf-related traits, petiole length, and plant height were the dominant factors contributing to morphological differentiation among the tested white clover genotypes.

Among the remaining traits, stolon number, stolon density, stolon length, and plant diameter contributed 11.58%, 10.79%, 8.84%, and 7.71%, respectively. Stolon number, stolon density, and stolon length were mainly associated with PC2, whereas plant diameter was mainly associated with PC1. The cumulative contribution of all nine traits reached 99.99%. Overall, these results indicate that leaf-related traits, petiole length, plant height, and stolon-related traits were the major contributors to morphological variation among global white clover genotypes (Table 2).

### 2.3. Correlation Analysis of Morphological Characters of the Tested White Clover Seed

**Figure 1:**
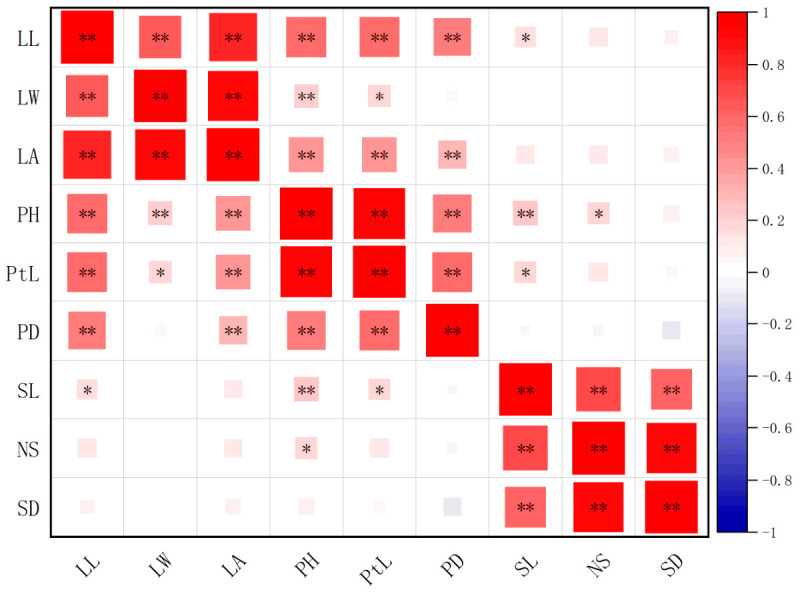
Correlation analysis of agronomic characteristics of 174 white clover samples. Abbreviations: LL: leaf length; LW: blade width; LA: leaf area; PH: plant height; PtL: petiole length; PD: plant diameter; SL: stolon length; NS: number of stolon segments; SD: stolon density. Numbers indicate Pearson correlation coefficients. ** Very significant correlation (*p* < 0.01) * Significant correlation (*p* < 0.05).

The correlation analysis results of 174 white clover accessions showed a significant correlation among morphological characteristics (Figure 1). A significant positive correlation among the six traits leaf length, leaf width, leaf area, plant height, petiole length, and plant diameter was observed. Extremely significant positive correlations among the three traits stolon length, the number of stolon nodes, and stolon density were recorded. The stolon length was positively correlated with leaf length, leaf width, leaf area, and plant height. Furthermore, the number of stolon nodes was positively correlated with plant height. The density of stolons showed non-significant correlations with leaf length, leaf width, leaf area, plant height, and petiole length, but was negatively correlated with plant diameter (Figure 1).

### 2.4. Cluster Analysis of White Clover Germplasm

**Figure 2:**
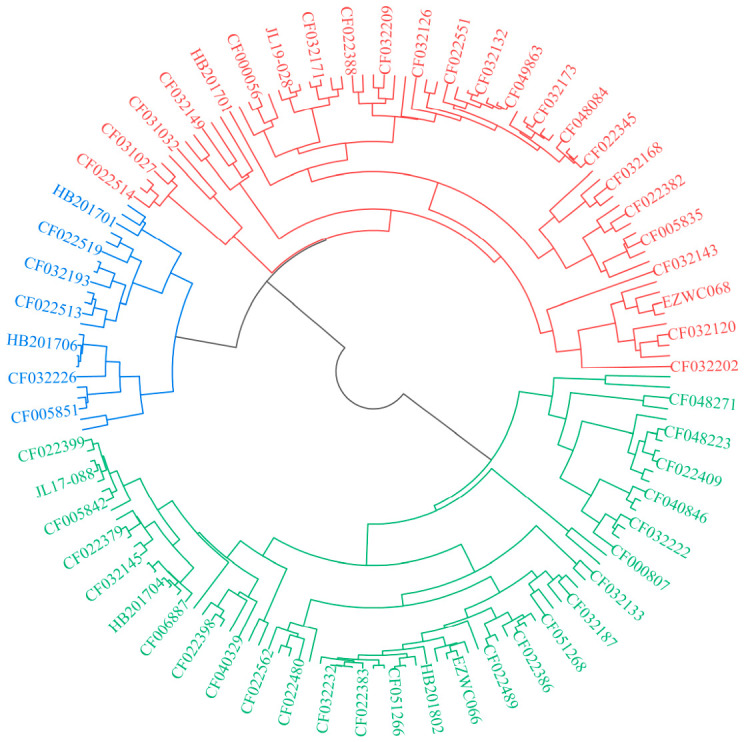
Cluster analysis of 174 white clover accessions based on 9 agronomic traits. The dendrogram reveals three major clusters (Groups I, II and III), distinguished by three colors: green represents Group I, red represents Group II, and blue represents Group III.

Based on clustering, all the germplasms were divided into three distinct groups, and the morphological characteristics of different groups are shown in Figure 2.

Group I consisted of 81 genotypes: 3 from South America, 3 from North America, 5 from Oceania, 38 from Europe, and 32 from Asia. The morphological indexes were plant height (13.69 ± 0.36) cm, leaf length (1.95 ± 0.06) cm, leaf width (2.12 ± 0.18) cm, leaf area (3.19 ± 0.83) cm^2^, and petiole length (12.43 ± 0.33) cm. The diameter of the plants was 1.65 ± 0.05 cm, the length of stolons was 10.69 ± 1.73 cm, the number of stolon nodes was 4.26 ± 0.88, and the density of stolons was 1.37 ± 0.27. This indicates that the plant is tall with large leaves, but has a moderate ability to spread horizontally.

Group II consisted of 70 accessions: 4 from South America, 3 from North America, 2 from Oceania, 35 from Europe, and 26 from Asia. Plant height was 11.71 ± 0.38 cm, leaf length 1.76 ± 0.04 cm, leaf width 1.67 ± 0.04 cm, leaf area 2.01 ± 0.13 cm^2^, petiole length 10.36 ± 0.31 cm, and plant diameter 1.29 ± 0.04 cm. The stolon length was 45.14 ± 3.33 cm, the number of stolon nodes 29.43 ± 2.92, and the stolon density 7.24 ± 0.55. This group of plants were relatively tall, with medium–large leaves and an extremely strong creeping ability.

Group III consisted of 23 varieties, including 1 from North America, 2 from Oceania, 7 from Europe and 13 from Asia. The plant height measured was 8.37 ± 0.4 cm, leaf length 1.56 ± 0.053 cm, leaf width 1.45 ± 0.05 cm with leaf area 1.28 ± 0.17 cm^2^, petiole length 7.61 ± 0.43 cm, and plant diameter 1.44 ± 0.06 cm. The stolon length was 14.39 ± 3.13 cm, number of stolon nodes 7.13 ± 1.66, and the density of stolons was 1.91 ± 0.43. This indicates that these plants have relatively short stems, small to medium-sized leaves and average spreading ability (Table 3).

Clustering was performed using the unweighted pair group method with arithmetic mean (UPGMA) based on Euclidean distance. The dendrogram revealed three major clusters (Groups I, II, III). Group I (*n* = 81) was characterized by tall plants and large leaves, Group II (n = 70) by strong stoloniferous growth (long stolons, high node density), and Group III (n = 23) by small, compact morphology.

### 2.5. Comprehensive Evaluation

In this study, the actual measurement values of agronomic traits of 174 white clover genotypes were standardized, and the scores of each germplasm in the three principal components were calculated. The proportion of the eigenvalue corresponding to the selected principal component to the sum of the three eigenvalues was taken as the weight, and the comprehensive F value of each germplasm material was calculated. The larger the F value, the better the comprehensive traits. The top 10 varieties recorded were CF051269, CF050015, CF022385, HB2017018, CF002737, XJ2016-93, CF032210, HB2017020 and CF022388. The top three were all from the first group, representing plant species that are tall and have larger leaves (Table 4).

### 2.6. Analysis of Photosynthetic Variation in Clover Genotypes

In order to evaluate the photosynthetic capacity of different germplasms, the photosynthetic parameters chlorophyll content and chlorophyll fluorescence of leaves were analyzed. The statistical analysis of nine physiological indicators of 174 white clover germplasms showed that there were significant differences among different genotypes. The net photosynthetic rate of the tested materials ranged from 4.01 to 18.46, with an average of 8.76. The stomatal conductance ranged from 0.22 to 1.40, with a mean of 0.59, the intercellular CO_2_ concentration ranged from 253.94 to 346.51, with an average of 297.09, and the transpiration rate measured was 2.06 to 9.08, averaging 5.25. Maximum photosynthetic efficiency ranged from 0.53 to 0.75, with an average of 0.70. Chlorophyll ranged from 1 to 5.61, with an average of 3.84. Chlb ranged from 0.8 to 5.72, with an average of 3.3. Total chlorophyll content ranged from 1.84 to 10.86, with a mean of 7.13, and the carotenoids ranged from 0.01 to 0.46, with an average of 0.14. The coefficient of variation of carotenoids was highest, 78.57%, followed by stomatal conductance, chlorophyll b, total chlorophyll content, and chlorophyll a: 40.68%, 38.79%, 34.22%, and 32.29%, respectively. The net photosynthetic rate and transpiration rate changed less, with coefficients of variation of 26.60% and 22.67%, respectively, while the intercellular carbon dioxide concentration and photosynthetic efficiency showed a little difference, with coefficients of variation of 4.84% and 4.29%, respectively (Table 5).

### 2.7. Principal Component Analysis of Photosynthesis in White Clover Germplasm

Principal component analysis was carried out on nine photosynthetic traits, and the results showed that the eigenvalues of the four principal components were all above 1.0. The cumulative contribution rate of the first four principal components reached 84.03%. Principal component 1 was 2.94 and the contribution rate was 32.65%. There were three characteristics with high absolute load values, which were chlorophyll a, chlorophyll b, and total chlorophyll content, with load values of 0.95, 0.93, and 0.97, respectively. These characteristics mainly reflected the relatively high chlorophyll content and strong photosynthetic capacity of germplasm materials. The eigenvalue of principal component 2 was 2, and the contribution rate was 22.3%. There were three traits with high absolute load values, which were net photosynthetic rate, stomatal conductance, and transpiration rate, with load values of 0.77, 0.70, and 0.82, respectively. The eigenvalue of principal component 3 was 1.48, and the contribution rate was 16.42%. There were two characteristics with high absolute load values, namely, intercellular CO_2_ concentration and stomatal conductance, whose load values were 0.92 and 0.57, which mainly reflected the light capacity of tested genotypes. The characteristic value of principal component 4 was 1.14, and the contribution rate was 12.66%. The characteristics with high load value showed maximum photosynthetic rate, with load values of 0.65 (Table 6).

### 2.8. Correlation Analysis of Photosynthesis in Experimental White Clover Germplasm

**Figure 3:**
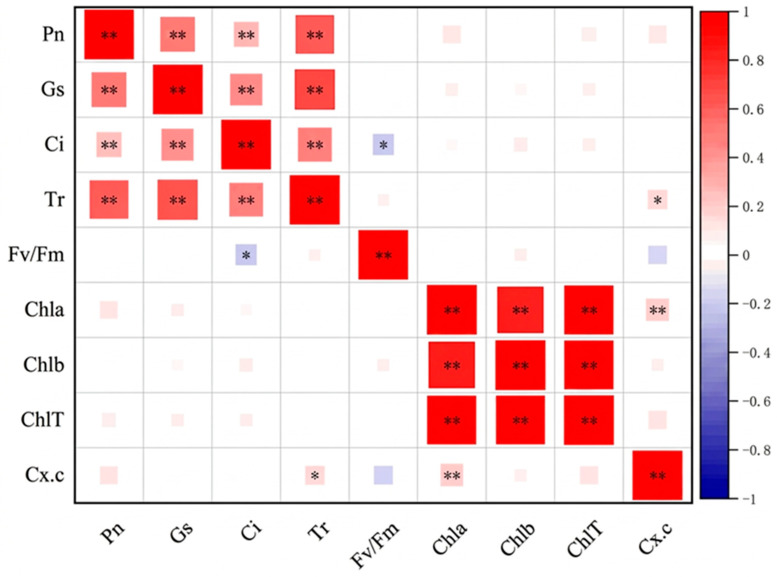
Correlation analysis of photosynthetic traits of 174 white clover. Abbreviations: Fv/Fm: maximum photosynthetic efficiency; Pn: net photosynthetic rate; Ci: intercellular CO_2_ concentration; Gs: stomatal conductance; Tr: transpiration rate; Chla: chlorophyll a; Chlb: chlorophyll b; ChlT: total chlorophyll content; Cx.c: carotenoids. Numbers indicate Pearson correlation coefficients; ** very significant correlation (*p* < 0.01); * significant correlation (*p* < 0.05). Red and blue indicate positive and negative correlations, respectively.

The results of 174 samples of white clover accessions showed that there were obvious correlations among individual indicators. The results showed that net photosynthetic rate was positively correlated with stomatal conductance, intercellular CO_2_ concentration, and transpiration rate. Stomatal conductance was positively correlated with intercellular CO_2_ concentration and transpiration rate. Chlorophyll a showed a positive correlation with chlorophyll b, total chlorophyll content, and carotenoids. There was a significant positive correlation between chlorophyll b and total chlorophyll content and carotenoids. A significant positive correlation between total chlorophyll content and carotenoids was observed (Figure 3).

### 2.9. SLAF-Seq Sequencing for Library Evaluation

The reads are enzymatically sliced segments of genomic DNA, and their base distribution will be affected by the enzyme cleavage site and PCR amplification. The first two bases of the sequenced reads will show the base separation consistent with the enzyme cleavage site, and the distribution of the subsequent bases will fluctuate to different degrees. E-enzymatic digestion was predicted for the reference genome of red clover, and according to the principle of enzyme digestion scheme selection, the restriction endonuclease digestion combination was determined as RsaI + HaeIII. Sequences with enzyme section lengths of 414–464 bp were defined as SLAF tags, which were predicted to yield 320,417 SLAF tags. Evaluation of control sequencing data was used to monitor whether the experimental process was normal or not and to determine the effectiveness of the implementation of the enzyme digestion program. In this experiment, 158.8 M data reads were obtained by sequencing the control genome, and the comparison results showed that the double-end comparison efficiency of this experiment (the comparison efficiency of two ends of a sequence on the reference genome) was basically normal. Enzymatic digestion efficiency is a key index to evaluate the success of simplified genome experiments. However, factors such as complex structural regions on the genome (such as ring structural domains, consecutive enzyme cleavage sites, etc.), low purity of individual genomic DNA, and insufficient enzyme cleavage time may affect the activity of restriction endonuclease, resulting in the fact that some of the enzyme cleavage sites are not cleaved [37]. By counting the proportion of residual cleavage sites in the inserted fragments of sequencing reads, a higher statistical proportion results represent better cleavage efficiency. The 95.25% double-end ratio efficiency in this experiment indicates that SLAF library construction is normal.

### 2.10. Sequencing Data Statistics

To ensure the quality of analysis, read length 126 bp × 2 was used as the subsequent data evaluation and analysis data in this study. A total of 2329.4 Mb read-length data were obtained from 174 white clover germplasm resources by sequencing on the Illumina HiSeq TM2500 sequencing platform. The number of read lengths of the different germplasm materials was in the range of 4,701,984–31,540,232. The Q30 of sequencing quality values of different materials ranged from 90.61% to 96.82%, with an average Q30 of 93.11%, and the Q30 of the control was 95.92%, indicating a low sequencing base error rate. The GC content obtained by sequencing ranged from 36.46% to 40.33%, with an average of 40.96%. The control GC content was 34.21%, which indicated that the GC content was low and met the sequencing requirements (Table 7).

### 2.11. Development and Identification of SLAF Tags and SNP Markers

Based on the sequencing results of 174 white clover individuals, a total of 320,417 SLAF tags were developed. The number of SLAF tags obtained from different white clover genotypes was inconsistent, basically ranging from 29,115 to 98,689, and the sequencing depths of each genotype varied greatly, with the total sequencing depths of the different accession ranging from 205,488 to 3,248,880, and the average depth of sequencing for each material ranging from 4.11% to 39.87%, with an average sequencing depth of 16.42. By typing all the SLAF tags, the polymorphic SLAF tags were finally obtained. The average sequencing depth of each material ranged from 4.11% to 39.87%, with an average sequencing depth of 16.42. By typing all SLAF tags, 202,625 polymorphic SLAF tags were obtained, accounting for 63.24% of the total number of SLAF tags. In addition, 2,999,555 SNP tags were obtained from polymorphic SLAF tags, and the number of SNP tags obtained from each genotype ranged from 1,118,565 to 2,056,614. The average completeness of these SNPs ranged from 37.29% to 68.56%, and the heterozygosity of SNPs ranged from 11.29% to 26.7%. The genetic fingerprinting profiles for all 174 accessions, including their geographic origin, genetic group assignment, and ADMIXTURE ancestry coefficients (Q1 and Q2), are presented in Supplementary Table S1. These combined genetic identifiers enabled the unique discrimination of each white clover accession in this study.

### 2.12. Population Genetic Structure and Genetic Evolution Analysis of White Clover Germplasm

#### 2.12.1. Genetic Structure Analysis

**Figure 4:**
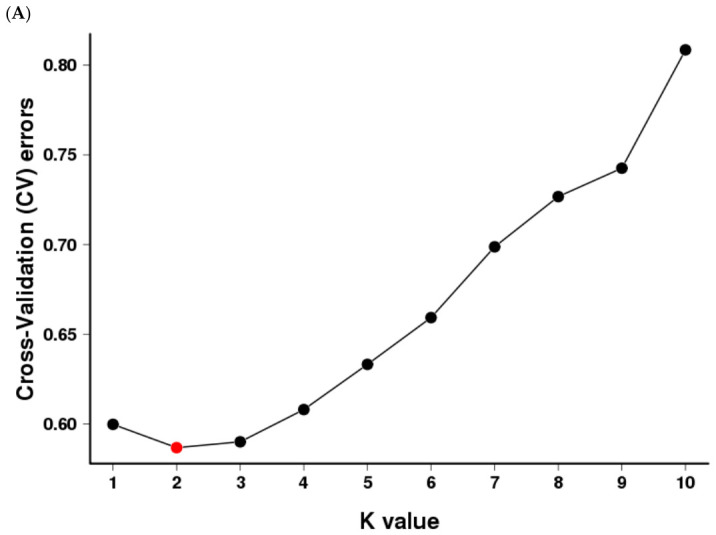

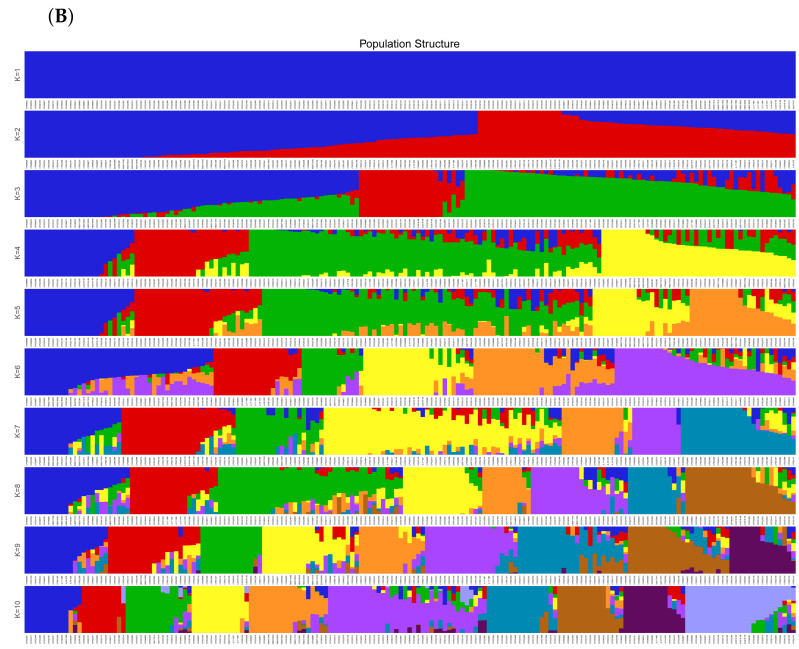
Population structure inferred by ADMIXTURE analysis. (**A**) Cross-validation error for different assumed numbers of ancestral populations (K = 1–10). The red point indicates the optimal K value, corresponding to the lowest cross-validation (CV) error. (**B**) ADMIXTURE bar plot for the selected model, K = 2. Each vertical bar represents one individual, and each color represents one inferred ancestral component. Therefore, increasing K increases the number of inferred ancestral populations and the number of colors shown. The additional ADMIXTURE plots for K = 1–10 illustrate how inferred ancestry proportions change under different assumed values of K.

The 174 white clover accessions were analyzed for population structure using the screened population SNP loci, the population structure was inferred for different K values (1–10), and the minimum CV error associated with each K value was calculated using Admixture software. The ADMIXTURE analysis showed that the lowest cross-validation error was observed at K = 2, indicating that the 174 white clover accessions mainly contained two ancestral genetic components. In contrast, the phylogenetic tree and PCA revealed further genetic differentiation among accessions and separated them into three genetic clusters, designated Groups I, II, and III. Therefore, the population structure was interpreted as two major ancestral backgrounds with three phylogenetic genetic subgroups. The three genetic groups were assigned mainly according to the phylogenetic tree topology and were further supported by PCA clustering patterns (Figure 4A,B).

#### 2.12.2. Evolutionary Analysis of Population Systems

**Figure 5:**
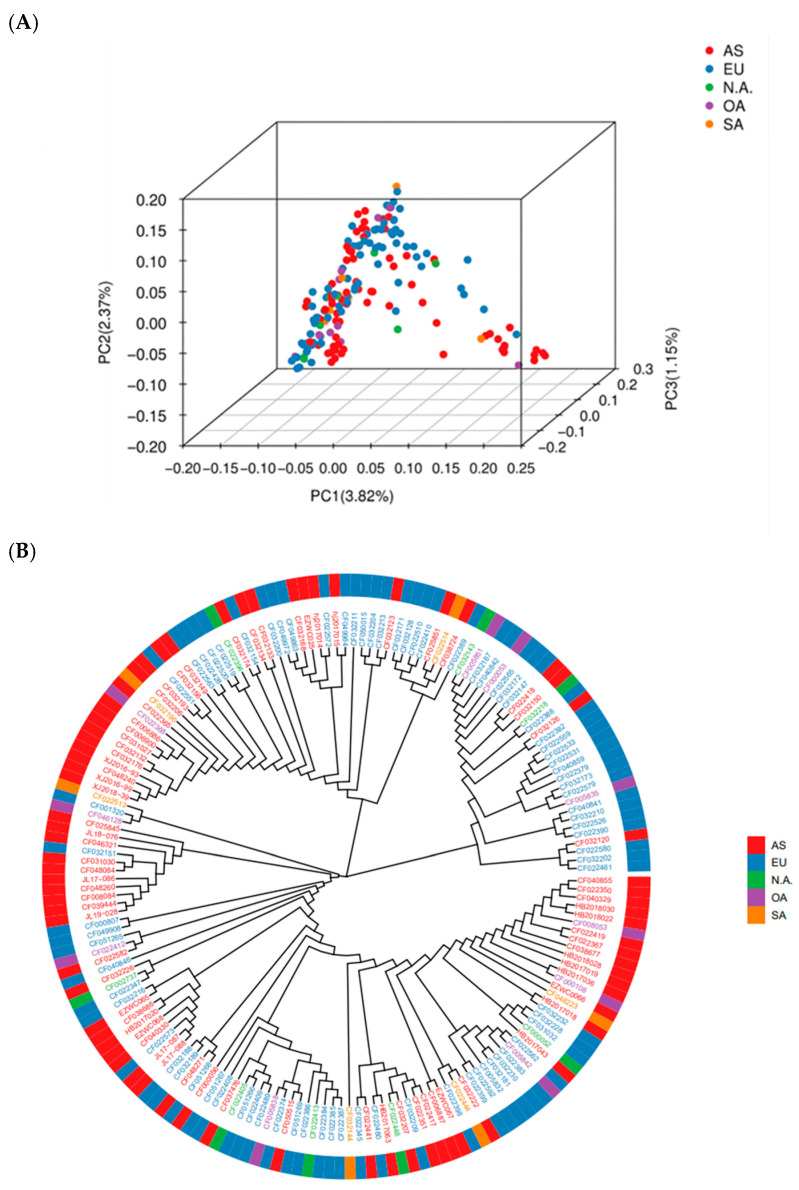
Genetic relationships among 174 white clover accessions. (**A**) Principal component analysis (PCA) of the same SNP dataset. PC1, PC2, and PC3 are shown. Colors indicate geographic origin: AS (Asia), EU (Europe), N.A. (North America), OA (Oceania), SA (South America). (**B**) Phylogenetic tree constructed using the neighbor-joining method based on 102,025 high-quality SNPs. Three major groups (I, II, III) are highlighted, despite STRUCTURE analysis supporting K = 2 (Figure 3), indicating further sub-structuring within one ancestral population.

Using cluster software (v3.0), principal component analysis (PCA) was conducted on 2,999,555 SNP loci to derive the principal component cluster analysis of white clover individuals. The three-dimensional clustering results (the first, second, and third principal components were PC1, PC2, and PC3, respectively) indicated a dense distribution of individuals across various sites on the PCA plot, revealing a significant degree of mixing (Figure 5A).

The phylogenetic tree of 174 white clover accessions revealed three major genetic groups (Figure 4B), despite the STRUCTURE analysis supporting K = 2. This suggests that one of the two ancestral populations has undergone further differentiation. Among them, Group I includes 30 individuals, of which 21 are in Europe, 4 in Asia, 3 in Oceania and 2 in North America, and this group is mainly distributed in Europe. Group II includes 47 resources, of which 25 genotypes are distributed in Asia, 20 in Europe, 2 in South America, and only one germplasm is in Oceania and North America, but most individuals were located in Asia and Europe. Group III included 97 resources, of which 40 varieties were distributed in Europe, 44 from Asia, 4 in South America and Oceania, and 5 in North America. This group mainly consisted of individuals in Asia and Europe. The results of germplasm clustering and morphological classification were basically the same (Figure 5B).

#### 2.12.3. Association Between Genetic Groups and Geographic Origin

To determine whether genetic groups are associated with geographic origin, one-way analysis of variance (ANOVA) and a chi-squared test was performed on the distribution of 174 accessions across three genetic groups (I, II, III) and five continents (Asia, Europe, North America, Oceania, South America). The chi-squared test revealed a significant association between genetic group and continent of origin (χ^2^ = 25.78, df = 8, *p* = 0.0012; Table S2). One-way ANOVA further confirmed a significant effect of genetic group on geographic distribution (F = 3.489, *p* = 0.032; Table S3). Post hoc Tukey HSD test revealed that Group I and Group III differed significantly (*p* = 0.028), while no significant differences were found between Group I and Group II (*p* = 0.148) or between Group II and Group III (*p* = 0.524).

Standardized residual analysis from the chi-squared test indicated that Group I was significantly underrepresented in Asia (residual = −2.35) and overrepresented in Europe (residual = +1.88), while Groups II and III showed more balanced distributions across continents. These results collectively indicate that the genetic clustering of white clover accessions is significantly correlated with their geographical provenance, with Group I showing a distinct European distribution pattern compared to Group III.

## 3. Discussion

This study combined high-throughput SLAF-seq genotyping with phenotypic evaluation to characterize the genetic, morphological, and physiological diversity of 174 white clover accessions. By integrating high-throughput SLAF-seq genotyping with detailed phenotyping for key agronomic and photosynthetic traits, we have unveiled the complex genetic architecture and population structure of this vital forage legume. Our findings confirm the existence of substantial diversity, which is not strictly partitioned by geographical origin, but is clearly reflected in distinct morphological and physiological groupings. This work establishes a critical foundation for the molecular-assisted breeding and efficient conservation of white clover genetic resources [38].

### 3.1. Morphophysiological Diversity and Its Implications for Breeding

The substantial variation observed in agronomic traits among the 174 white clover accessions demonstrates that this germplasm panel contains rich phenotypic diversity and has considerable potential for breeding improvement. As shown in Table 1, the coefficients of variation for agronomic traits ranged from 24.59% to 139.02%, indicating a high level of morphological diversity among the tested genotypes. This wide variation reflects substantial differences in plant architecture, leaf morphology, and stolon development. Such diversity is essential for breeding because it provides the phenotypic basis for selecting accessions with contrasting and complementary characteristics, such as large leaves, strong stoloniferous ability, high biomass potential, and improved persistence. The cluster analysis based on manifestations of nine morphological traits also delineated three groups (Figure 2), which intriguingly aligned with the genetic clusters and were characterized by distinct growth strategies. Group I (81 accessions) represents individuals with an upright growth habit, characterized by taller plant height, larger leaves, and moderate stolon development. This morphology is typical of ladino or large-leaved types selected for high biomass production under cutting or lax grazing regimes [4]. The top-ranked varieties from our comprehensive evaluation (e.g., CF051269, CF050015) (Table 4) belonged to this group, identifying them as excellent material for forage yield breeding programs. Group II (70 accessions) is defined by its exceptionally strong stoloniferous capacity, featuring long stolons with high node density. This “runner-type” morphology is a key adaptation for persistence under intensive grazing, as it allows plants to rapidly colonize bare patches and withstand defoliation [20]. The positive correlation between stolon traits and plant height in this group suggests a synergistic growth strategy for both persistence and productivity. Group III (23 accessions) comprises smaller, more compact plants with reduced leaf size and moderate spreading ability. These germplasms likely represent ecotypes adapted to resource-limited environments or heavy grazing pressure, where a low-growth habit is advantageous for survival.

The strong positive correlations among leaf-related traits (LL, LW, LA), plant height and petiole length (PH, PtL), and among stolon-related traits (SL, NS, SD) indicate that these sets of traits are co-inherited and can be selected for concurrently (Figure 1). The independence of these trait complexes, as shown by the separate principal components, provides breeders with the flexibility to pyramid different combinations, e.g., selecting large leaves within a highly stoloniferous background.

The principal component analysis of morphological traits further clarified the contribution of different agronomic traits to phenotypic diversity (Table 2). The principal components separated the measured traits into different functional modules, mainly associated with leaf size, plant architecture, and stolon development. This result is consistent with the correlation patterns shown in Figure 1, indicating that white clover morphological diversity is shaped by multiple trait complexes rather than by a single dominant trait.

In addition to agronomic traits, photosynthetic traits also showed considerable variation among the 174 accessions (Table 5). The coefficients of variation for photosynthetic traits ranged from 4.29% to 78.57%, suggesting that some physiological traits were relatively stable across the germplasm panel, whereas others showed substantial diversity and selection potential. The variation among photosynthetic indicators was considerable in pigment content (Chla, Chlb, carotenoids) and stomatal conductance (Gs). The high variability in carotenoids, which are involved in photoprotection, suggests that some accessions may possess enhanced resilience to photooxidative stress [39]. The principal component analysis separated the photosynthetic traits into distinct functional modules: pigment content, gas exchange parameters (Pn, Gs, Tr), and photochemical efficiency (Fv/Fm, Ci). The high heritability of Fv/Fm across all germplasms suggests that the maximum quantum efficiency of PSII is a stable and genetically controlled trait in this panel (Table 6), in line with [40]. The correlation between net photosynthetic rate (Pn) and stomatal conductance (Gs) underscores the role of stomatal regulation in limiting carbon assimilation, a factor crucial for drought tolerance (Figure 3) [21]. The germplasms with high pigment content coupled with high photosynthetic rates identified here are valuable resources for enhancing the physiological efficiency and productivity of new cultivars. Nevertheless, the high CV values observed for certain photosynthetic parameters indicated that physiological traits also contain exploitable variation for breeding. Therefore, the combined evaluation of agronomic and photosynthetic traits provides a more comprehensive basis for the identification of excellent germplasm.

### 3.2. Decoupling of Genetic Structure from Geographical Origin

The population genetic analyses revealed detectable genetic structuring among the 174 white clover accessions. Based on the high-quality SNP dataset generated in this study, the ADMIXTURE analysis indicated two main ancestral components (K = 2; Figure 4), suggesting that the tested accessions were not genetically homogeneous. The SNP-based principal component analysis further illustrated the genetic relationships among accessions and showed partially overlapping distributions among groups (Figure 5A). The chi-squared test, χ^2^ = 25.78, *p* value = 0.0012, and one-way ANOVA, F statistic = 3.489, *p* value = 0.032, suggested a possible association between genetic grouping and geographic origin. However, this association should be interpreted carefully because the accessions were unevenly distributed among geographic regions and some continents were represented by relatively few samples. Post hoc analysis indicated that Group I (predominantly European) is significantly different from Group III (widely distributed across Asia and Europe), while Group II shows an intermediate distribution pattern. Each of the three genetic groups contained germplasms from multiple continents, with Europe and Asia being widely represented in all groups. Among them, the top three varieties, CF051269, CF050015, and CF022385, all belong to the first category group. Their plants and leaves are relatively large, and they can be used for the morphological improvement of white clover. The broad geographic distribution of accessions within the same genetic groups may be partly related to historical and modern germplasm exchange, including seed trade, exchange among breeding programs, and intentional introduction of germplasm into new environments [2]. Similar patterns of geographically unstructured genetic diversity have been observed in other widely distributed forage species, reflecting their allogamous mating system and the globalized nature of pasture improvement [26]. The statistical association between genetic groups and geographic origin should therefore be interpreted together with the observed admixture and PCA patterns. The contingency table (Supplementary Table S2) and ANOVA results (Supplementary Table S3) provide limited evidence for a possible relationship between geographic origin and genetic grouping, whereas the ADMIXTURE and PCA results (Figure 4 and Figure 5A) show that the genetic structure is also characterized by admixture and overlapping distributions. This combined evidence suggests that conservation and breeding programs should not rely solely on geographic information. Instead, molecular characterization should be used to identify genetically distinct accessions and to avoid redundancy in germplasm conservation.

### 3.3. High-Throughput SNP Discovery and Robust Genotyping via SLAF-Seq

The successful application of SLAF-seq in white clover, a species without a fully sequenced reference genome, underscores the utility of this reduced-representation sequencing approach for genetic diversity studies in non-model crops [29,31]. The SLAF-seq analysis provided a genomic perspective that largely corroborated the phenotypic findings. The development of 2,999,555 high-quality SNP markers from 202,625 polymorphic SLAF tags confirms the high genetic diversity at the DNA level, consistent with the observed phenotypic variation. Our sequencing output was robust, with high average quality scores (Q30 > 93%) and a total of 320,417 developed SLAF tags (Table 7). The high polymorphism rate (63.24% of SLAF tags) and the subsequent identification of over 2.9 million SNPs demonstrate the high level of genetic variation present within the global white clover germplasm. This vast repository of SNP markers represents a significant genomic resource, far surpassing the capacity of traditional markers, and enables a high-resolution analysis of population structure and genetic relationships [41]. In addition to population genetic analysis, the SNP dataset supported the development of molecular fingerprint information for the tested accessions (Table S1). This supplementary fingerprint pattern (File S1) provides accession-specific molecular identifiers that can be used for germplasm identification, variety authentication, and management of breeding materials.

### 3.4. Integration of Phenotypic and Genomic Data for Future Breeding

The congruence between the morphological and molecular classifications is remarkable and reinforces the biological relevance of the identified genetic groups. It demonstrates that the SLAF-seq-derived SNPs effectively capture the genetic underpinnings of visible, agronomically important traits [42]. This successful integration paves the way for genome-wide association studies (GWASs) using the high-density SNP map generated in this study. The identified trait–marker associations can be used to develop molecular markers for marker-assisted selection (MAS), accelerating the breeding cycle for complex traits like stolon density, leaf size, and photosynthetic efficiency [24,25]. For instance, breeders could rapidly introgress the high-stolon-density alleles from Group II germplasms into the high-yielding genetic background of Group I to develop persistent and productive cultivars. Furthermore, the genetic diversity characterized here provides a buffer against environmental stresses, including pests, diseases, and climate change. The diverse germplasm, particularly those in Group III, which may harbor stress-adaptive alleles, can be utilized to enhance the resilience of modern white clover cultivars [18]. By integrating these datasets, breeders can select germplasm that is both phenotypically superior and genetically diverse, enabling more efficient trait pyramiding, genome-wide association analysis, and marker-assisted selection. This integrated approach will support the development of white clover cultivars with improved productivity, persistence, photosynthetic efficiency, and environmental resilience.

## 4. Materials and Methods

### 4.1. Sample Collection and Growth Conditions

**Figure 6:**
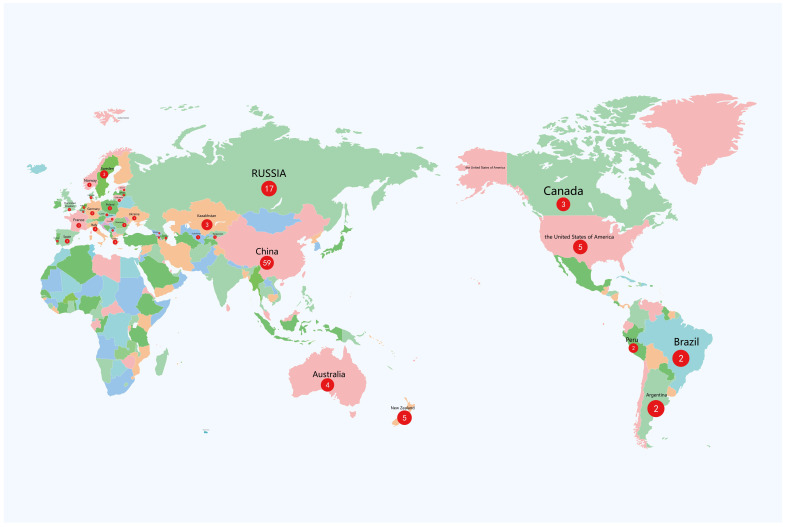
Germplasm resources of 174 white clover samples worldwide.

**Figure 7:**
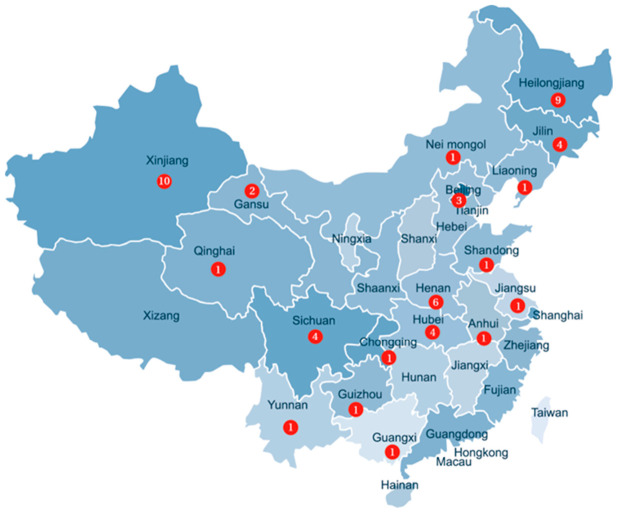
Germplasm resources of 55 white clover samples from China.

**Figure 8:**
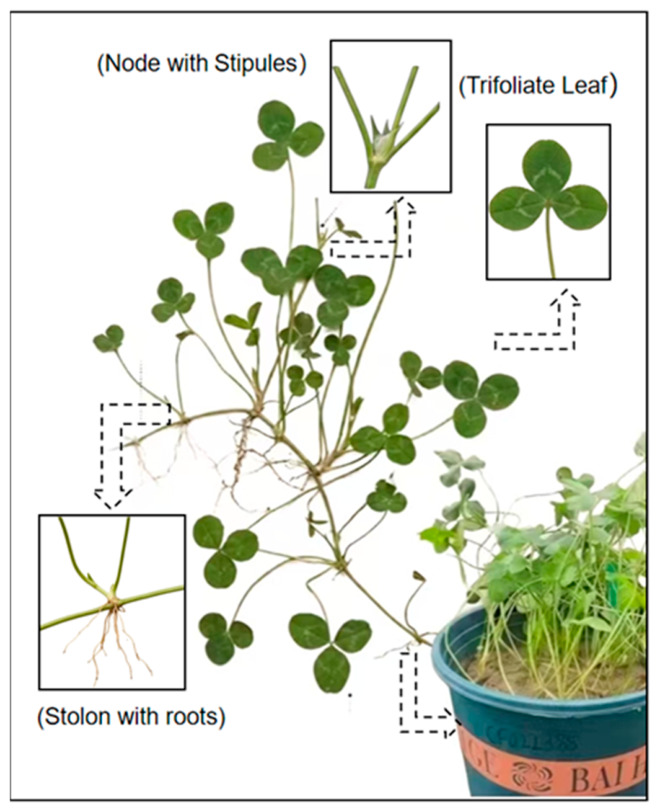
Morphological presentation of white clover (*Trifolium repens* L.).

A total of 174 white clover (*Trifolium repens* L.) accessions (Table 8) were obtained from the National Herbage Germplasm Bank of China, Beijing. The material was mainly distributed in 39 countries across five continents: Europe, Asia, Oceania, South America, North America. Europe: 79 genotypes distributed in 25 countries, of which Russia had 17, accounting for the largest proportion; Asia: 71 accessions were distributed in 7 countries, of which 55 were from China, accounting for the largest proportion; Oceania: 9 germplasms were distributed in 2 countries, and 5 genotypes were tested in Australia and New Zealand; North America: 8 genotypes, distributed in 2 countries, Canada and the United States, of which the United States had the majority, with 5 varieties; South America: 7 germplasms from 3 countries: Argentina, Brazil, and Peru (Figure 6 and Figure 7 below). The experiment was conducted in the greenhouse of the Wenhui Road Campus of Yangzhou University, Jiangsu, China (32°20′ N, 119°23′ E) at 25 °C with a 12/12 h photoperiod. On 12 April 2023, 15 seeds of each genotype were grown in plastic pots (diameter of 17.5 cm and a height of 16 cm). Thus, each accession was represented by one individual pot. All pots were randomly arranged in the greenhouse to minimize positional effects. Field soil without any additional substrate or amendment was used for all pots to reduce variation caused by soil conditions. To avoid developmental variation among accessions, phenotypic and photosynthetic traits were measured at the same developmental stage. Measurements were conducted at the vegetative growth stage, corresponding to BBCH stage 39. For each accession, fully expanded and healthy trifoliate leaves were selected from representative plants for photosynthetic measurements. Photosynthetic traits were measured between 8:00 and 11:00 on sunny days. Multiple healthy plants within each pot were measured as individual-level subsamples rather than independent pot-level biological replicates, and the mean value was used to represent the phenotypic performance of each genotype in subsequent analyses. To provide a clear morphological reference for the studied taxon (*Trifolium repens* L.), a detailed photograph is presented in Figure 8.

### 4.2. Morphological Indicators

A total of 9 morphological indexes were measured—plant height, leaf length, leaf width, leaf area, petiole length, plant diameter, stolon length, stolon number, and stolon density—following the method of Weith [43]. The measurements were conducted at the flowering stage. Briefly, six healthy individual plants were randomly selected from each accession and the average value was calculated.

The measured plant’s leaves, internodes, and sampling areas were considered subsamples within each accession rather than independent biological replicates. Because the number of measured individuals was lower than that commonly recommended for comprehensive morphological evaluation, the morphological data were used primarily for preliminary and descriptive accession-level phenotypic characterization under greenhouse conditions. Plant height (cm) was measured from the ground to the top of the plant. For leaf length and leaf width (cm), mature leaves were randomly selected from 6 different clusters of each genotype to measure the length (petiole to leaf tip) and width (widest part of leaf) of 6 leaflets. The leaf area (cm^2^) was calculated by the formula y = −1.8467 + 0.337X_1_ + 1.9705X_2_ − 0.568X_3_ + 0.6292X_4_ (X_1_ is the leaf length, X_2_ is the leaf width, X_3_ is the leaf length + leaf width, X_4_ is the leaf length × leaf width) [44]. Petiole length (cm) was measured as the length of mature axillary petioles. Stolon length (cm) was measured from the base to the terminal bud of selected stolons bearing leaves. For stolon number, 12 randomly selected internodes corresponding to the measured stolon lengths were averaged. Stolon density was calculated as the number of stolons within a 10 cm × 10 cm area at the center of each plant, averaged from 12 randomly selected plants.

### 4.3. Photosynthesis-Related Indicators

Photosynthetic indexes such as net photosynthetic rate (Pn), stoma conductivity (Gs), intercellular CO_2_ concentration (Ci), and transpiration rate (Tr) were measured using an LI-6400XT portable photosynthesis system (Li-Cor Biosciences, Lincoln, NE, USA) as per ref. [45]. For each measurement, 4–5 joining leaves (the second fully unfolded leaf from the top) were inserted into the chamber between 8:00 and 10:00 AM. Measurements were made at a light intensity of 1000 μmol photon m^−2^ s^−1^ and a constant airflow of 500 μmol s^−1^. Each measurement was repeated three times to calculate the average.

The maximum quantum efficiency (Fv/Fm) of the photosystem II and chlorophyll fluorescence parameters were determined as per Maxwell (2000) [46] using the same portable chlorophyll fluorometer (FluorPen FP 110/D; Photon Systems Instruments, Drásov, Czech Republic) at the end of the experiment in greenhouse conditions between 9:00 and 11:00 AM. Fully expanded healthy leaves at the same position were selected for measurement. Before measurement, leaf samples were placed in the dark for 20 min to make them dark-adapted. The fluorometer was operated for 2 s, the emission wavelength used was 650 nm, the intensity 3500 μmol photon m^−2^ s^−1^, and the minimum fluorescence F_o_ and maximum fluorescence F_m_ of the dark-adapted leaves were measured. Dark adaptation and light adaptation initial fluorescence (F_o_ and F_o_′) of the blade surface were measured under PPFD modulation irradiation less than 0.1 μmol m^−2^ s^−1^. For each treatment, 3 biological replicates and 6 leaves per replicate were measured. The formula for calculating chlorophyll fluorescence parameters is: Fv/Fm= [(Fm − Fo)/Fm].

To determine the chlorophyll content, white trifoliate leaves in a similar growth state were selected. Pigment contents were measured using ethanol extraction as per Lichtenthaler (1987) [47]. In short, 0.3 g of leaf samples were weighed out and 3 mL of 95% ethanol and a small amount of quartz sand and calcium carbonate added to prepare a homogenate. This was filtered into a volume bottle, the mortar washed with 95% ethanol and filtered until the filter paper became colorless, and the volume was fixed at 25 mL. After dark treatment of 24 h, 200 μL extract solution was added into a 96-well plate, and the absorption value was determined at 470 nm, 649 nm, and 665 nm. The calculation formulas are as follows.

Chla (mg/L) = 13.95A665 − 6.88A649

Chlb (mg/L) = 24.96A649 − 7.32A665

ChlT (mg/L) = 18.16A649 + 6.63A665

Cx.c (mg/L) = (1000A470 − 2.05Ca − 114.8Cb)/245

### 4.4. DNA Sampling, Extraction, and Quality Testing

For DNA extraction, young and healthy trifoliate leaves of 3–5 randomly selected plants per genotype at the vegetative growth stage were selected. Equal amounts of leaf tissue (about 0.2 g) from these plants were collected, and the sampled leaves were not used for subsequent chlorophyll or photosynthetic measurements. Therefore, DNA sampling did not affect the phenotypic evaluation. The collected samples were immediately frozen in liquid nitrogen and stored at −80 °C until DNA extraction. The whole DNA content of white clover germplasm was isolated using the 3×CTAB method. Subsequently, the quantity and integrity of the DNA were assessed through electrophoresis on 1% agarose gel to confirm compliance with the standards necessary for library construction. DNA concentration and quality were determined using a Nanodrop ND-1000 spectrophotometer (Thermo Fisher Scientific, Wilmington, DE, USA).

### 4.5. SLAF Library Construction and High-Throughput Sequencing

One hundred and seventy-four accessions of *Trifolium repens* from 39 countries spanning 5 continents underwent SLAF-seq. The experimental program was systematically designed through bioinformatic analysis, utilizing the *Trifolium pratense* genome as a reference (version Tp1.0, genome size ~158.8 Mb, published by Osterman J et al., 2021) [48]. Following high-throughput sequencing, a substantial volume of sequences was processed to identify polymorphic SLAF tags through software alignment, enabling the identification of specific SNP site distributions. A total of 320,417 SLAF tags were generated, comprising 202,625 polymorphic SLAF tags. Sequence analysis led to the discovery of 2,999,555 SNPs. Subsequently, 102,025 high-consistency SNP groups were identified by applying selection criteria of integrity > 0.5 and MAF > 0.05. Phylogenetic tree construction, population structure analysis, and PCA were conducted on the 174 white clover individuals based on the refined SNPs using statistical methods to elucidate genetic differentiation relationships at the genomic level.

Illumina HiSeq TM2500 (Illumina, Inc., San Diego, CA, USA) was employed to perform double-end sequencing of the qualified library. Following sequencing, clean sequences were generated through de-linking, removal of low-quality reading frames, and decontamination procedures. *Trifolium pratense* served as a control, and the accuracy and validity of the library construction were assessed based on alignment efficiency, restriction enzyme digestion efficiency, and insert length distribution. Ultimately, data quality was evaluated by calculating GC content and Q30 [49].

### 4.6. Design of Enzymatic Cutting Scheme

At the time of the experiment, library design and initial analysis (early 2023), a high-quality, haplotype-resolved white clover reference genome was not widely available. Therefore, we used the *Trifolium pratense* reference genome (version Tp1.0, ~158.8 Mb, Osterman J et al., 2021) as a proxy for restriction enzyme digestion simulation [48]. Due to the absence of published genome sequence data of white clover, the reference genome *Trifolium pratense*: Tp1. The RsaI + HaeIII restriction enzyme combination was employed. The resulting restriction fragments were modified with A at the 3′ end, linked with a dual-index sequencing adapter, and subsequently subjected to PCR amplification, purification, sample pooling, and gel excision to isolate the desired experimental fragments.

### 4.7. Development of SLAF Tags and SNP Markers

The raw data obtained from sequencing were identified using a dual index to obtain reads (segregating alleles) for each individual. After filtering the joints of sequencing reads, the quality of sequencing and the amount of data were evaluated. The control data were used to evaluate the efficiency of RsaI + HaeIII cleavage to determine the accuracy and validity of the experimental process. The reads generated from the sequencing of this experiment were from the same or similar lengths of sections produced by the same restriction endonuclease in different locations of white clover. The reads from 10 individuals were clustered according to the similarity of the sequences, and the reads that were clustered together originated from the same SLAF tags. The sequence similarity of the same SLAF tag among different individuals is much higher than that among different SLAF tags. The existence of sequence differences (i.e., polymorphism) among different individuals in the same SLAF tag can be defined as a polymorphic SLAF tag. The sequence with the highest depth in each SLAF tag was used as the reference sequence to develop genome-wide single-nucleotide polymorphism (SNP) markers, and the developed SNPs were screened according to the criteria of completeness > 0.5 and MAF > 0.05. Completeness > 0.5 indicates that the SNP locus was successfully genotyped in more than 50% of the individuals, corresponding to a missing rate < 50%; therefore, SNP loci with a missing rate ≥ 50% were removed. In addition, SNP loci with excessive heterozygosity, heterozygosity > 80%, were removed before downstream analysis. The representative high-quality SNPs were used to conduct genetic evolutionary tree analysis, genetic structure analysis, and principal component analysis (PCA). To distinguish true allelic SNPs from homoeologous variation arising from the allotetraploid nature of white clover (2n = 4x = 32), we applied the following stringent filtering criteria: (i) only SLAF tags with a sequencing depth ≥ 10× were retained; (ii) SNPs with more than two alleles in any individual were discarded as potential homoeologous sites; (iii) SNPs showing excessive heterozygosity (>80%) across all accessions were also removed, as they likely represented fixed differences between sub-genomes; and (iv) only biallelic SNPs with minor allele frequency (MAF) > 0.05 and completeness > 0.5 were retained for downstream analysis. This filtering strategy has been validated in previous SLAF-seq studies in polyploid species [29,42].

### 4.8. Data Analysis and Statistical Methods

SPSS (v20.0) software was used for variance analysis, correlation analysis, principal component analysis, and Euclidean distance cluster analysis of morphological and photosynthetic traits. Pearson’s correlation analysis was performed to evaluate the relationships among agronomic and photosynthetic traits. The significance of correlations was determined using two-tailed tests. Correlations were considered significant at *p* < 0.05 and highly significant at *p* < 0.01. The correlation heatmap was generated using Origin 2022 software, with correlation coefficients and significance levels displayed in the figure. Hierarchical clustering of phenotypic traits was performed using the unweighted pair group method with arithmetic mean (UPGMA) based on Euclidean distance, as implemented in SPSS (v20.0). Coefficients of variation (CV%) were analyzed by the formula CV (%) = δ/μ × 100%, where δ is standard deviation and μ is average value, calculated by Excel 2007 software for morphological and photosynthetic indicators. Based on SNP-labeled data, a phylogenetic tree was created using MEGA-CC software (MEGAX) (ver. 6.0) [50]. ADMIXTURE (v1.22) was employed to construct population genetic structure and PCA was performed using a smart PCA program to obtain the clustering of the samples from EIGENSOFT (v6.0) software [51]. In this study, ADMIXTURE analysis supported K = 2, indicating that the 174 white clover accessions mainly contained two ancestral genetic components. However, the phylogenetic tree and PCA revealed further genetic differentiation among the accessions and separated them into three genetic clusters. Therefore, ADMIXTURE was used to infer the major ancestral components, whereas genetic group assignments were determined primarily according to the topology of the phylogenetic tree and further supported by PCA clustering patterns. The three genetic clusters were designated Groups I, II, and III. DNA fingerprinting of the 174 white clover accessions was constructed based on the ADMIXTURE ancestry coefficients (Q matrix) and genetic group assignments derived from the phylogenetic analysis. For each accession, the ancestry proportions (Q1 and Q2, representing membership coefficients for the two inferred ancestral populations, K = 2) and the genetic group (I or II) were used as fingerprinting identifiers. To assess the relationship between genetic groups and geographic origin, a chi-squared test was performed to examine the association between the phylogenetic group and continent of origin. Statistical analyses were performed using the corresponding software described above.

## 5. Conclusions

Our study focused on the power of SLAF-seq to dissect the genetic diversity and population structure of a global white clover collection. The three genetic groups show significant association with geographic origin (χ^2^ = 25.78, *p* = 0.0012; ANOVA: F = 3.489, *p* = 0.032). Group I is predominantly found in Europe, Group II across Asia and Europe, while Group II occupies an intermediate space. The consistency between phenotypic clustering (based on agronomic traits) and molecular clustering (based on SNP data) suggests that our SNP dataset captures biologically meaningful genetic variation, providing a solid foundation for future genome-wide association studies (GWASs) and marker-assisted selection (MAS) in white clover. This resource provides an invaluable toolkit for germplasm conservation, enabling the identification of unique and complementary accessions to maintain a diverse gene pool. More importantly, it empowers molecular breeding initiatives aimed at developing high-yielding, persistent, and climate-resilient white clover varieties for sustainable agriculture. Future work will focus on conducting GWASs using the SNP dataset generated in the current study to pinpoint the precise genomic regions controlling the key agronomic and photosynthetic traits characterized here.

## Figures and Tables

**Table 1:** Variability analysis of agronomic traits of 174 white clover plants.

Characteristics	Min	Max	Average	Standard Deviation	CV	F
LL	1.25	4.94	1.83	0.45	24.59%	9.92
LW	0.98	6.9	1.79	0.72	40.22%	13.1
LA	0.13	28.13	2.47	2.8	113.36%	12.29
PH	5.90	24.13	12.19	3.52	28.88%	9.53
PtL	5.45	22.68	10.96	3.17	28.92%	6.11
PD	0.75	3.21	1.48	0.41	27.70%	0.08
SL	0.00	150.51	25.04	26.92	107.51%	280.45
NS	0.00	146.00	14.76	20.52	139.02%	162.43
SD	0.00	30.00	3.80	4.43	116.58%	108.18

Abbreviations: LL: Leaf length; LW: blade width; LA: leaf area; PH: plant height; PtL: petiole length; PD: plant diameter; SL: stolon length; NS: number of stolon segments; SD: stolon density; CV, coefficient of variation; F, F-statistic from analysis of variance.

**Table 2:** Principal component analysis of morphological traits.

Characteristic	Overall Contribution Across PC1–PC3 (%)	Cumulative Contribution (%)	Main Associated Component
LA	12.32	12.32	PC1
LW	12.22	24.54	PC1
PtL	12.19	36.73	PC1
LL	12.18	48.91	PC1
PH	12.16	61.07	PC1
NS	11.58	72.65	PC2
SD	10.79	83.44	PC2
SN	8.84	92.28	PC2
PD	7.71	99.99	PC1

Abbreviations: LL: leaf length; LW: blade width; LA: leaf area; PH: plant height; PtL: petiole length; PD: plant diameter; SN: stolon number; NS: number of stolon segments; SD: stolon density. Contribution (%) indicates the overall contribution of each morphological trait across PC1–PC3. The overall contribution was calculated using the sum of squared loadings. Main associated component indicates the principal component with the highest absolute loading for each trait.

**Table 3:** Averages of agronomic traits in different groups.

Group	LL	LW	LA	PH	PtL	PD	SL	NS	SD
I	1.95 ± 0.06	2.12 ± 0.18	3.19 ± 0.83	13.69 ± 0.36	12.43 ± 0.33	1.65 ± 0.05	10.69 ± 1.73	4.26 ± 0.88	1.37 ± 0.27
II	1.76 ± 0.04	1.67 ± 0.04	2.01 ± 0.13	11.71 ± 0.38	10.36 ± 0.31	1.29 ± 0.04	45.14 ± 3.33	29.43 ± 2.92	7.24 ± 0.55
III	1.56 ± 0.053	1.45 ± 0.05	1.28 ± 0.17	8.37 ± 0.4	7.61 ± 0.43	1.44 ± 0.06	14.39 ± 3.13	7.13 ± 1.66	1.91 ± 0.43

Abbreviations: LL: leaf length; LW: blade width; LA: leaf area; PH: plant height; PtL: petiole length; PD: plant diameter; SL: stolon length; NS: number of stolon segments; SD: stolon density.

**Table 4:** Top 10 rankings of the comprehensive evaluation of white clover varieties.

Comprehensive Evaluation	Group	F Value	Ranking
CF051269	I	9.143	1
CF050015	I	7.878	2
CF022385	I	4.943	3
HB2017018	II	4.702	4
CF002737	II	4.364	5
CF022367	I	4.005	6
XJ2016-93	I	3.981	7
CF032210	III	3.925	8
HB2017020	II	3.391	9
CF022388	II	2.528	10

Abbreviations: F: comprehensive evaluation score calculated as the weighted sum of principal component scores, with weights determined by the contribution rate of each principal component. A higher F value indicates better overall performance of the accession based on the evaluated traits.

**Table 5:** Variation in photosynthetic traits.

Characteristics	Min	Max	Average	Standard Deviation	CV
Pn	4.01	18.46	8.76	2.33	26.60%
Gs	0.22	1.40	0.59	0.24	40.68%
Ci	253.94	346.51	297.09	14.39	4.84%
Tr	2.06	9.08	5.25	1.19	22.67%
Fv/Fm	0.53	0.75	0.70	0.03	4.29%
Chla	1.00	5.61	3.84	1.24	32.29%
Chlb	0.80	5.72	3.30	1.28	38.79%
ChlT	1.84	10.86	7.13	2.44	34.22%
Cx.c	0.01	0.46	0.14	0.11	78.57%

Abbreviations: Fv/Fm: maximum photosynthetic efficiency; Pn: net photosynthetic rate; Ci: intercellular CO_2_ concentration; Gs: stomatal conductance; Tr: transpiration rate; Chla: chlorophyll a; Chlb: chlorophyll b; ChlT: total chlorophyll content; Cx.c: carotenoids; CV (%): coefficient of variation.

**Table 6:** Principal component analysis of photosynthesis in white clover accessions.

Characteristic	Overall Contribution Across PC1–PC3 (%)	Cumulative Contribution (%)	Main Associated Component
ChlT	14.08	14.08	PC1
Chla	13.51	27.59	PC1
Chlb	12.94	40.53	PC1
Ci	12.67	53.2	PC3
Gs	12.2	65.4	PC2/PC3
Tr	10.06	75.46	PC2
Fv/Fm	9.34	84.8	PC4
Pn	8.87	93.67	PC2
Cx.c	6.32	99.99	PC4

Abbreviations: Fv/Fm: maximum photosynthetic efficiency; Pn: net photosynthetic rate; Ci: intercellular CO_2_ concentration; Gs: stomatal conductance; Tr: transpiration rate; Chla: chlorophyll a; Chlb: chlorophyll b; ChlT: total chlorophyll content; Cx.c: carotenoids. Values with an absolute load less than 0.5 are not displayed. Contribution (%) indicates the overall contribution of each morphological trait across PC1–PC3. The overall contribution was calculated using the sum of squared loadings. Main associated component indicates the principal component with the highest absolute loading for each trait.

**Table 7:** Statistics of sequencing data.

Sample	Total Read Number (Mb)	Q30 Percentage	GC Percentage
174 accessions of *T. repens*	2329.40	93.11	40.96
*Trifolium pratense*	158.80	95.92	34.21

Abbreviations: GC percentage (%) represents the proportion of guanine (G) and cytosine (C) bases in the total sequencing reads.

**Table 8:** Origin and materials of white clover.

**No.**	**Genotypes**	**Sampling Site**	**No.**	**Genotypes**	**Sampling Site**
1	CF000052	North America, Canada	26	CF022382	Europe, Hungary
2	CF000053	Oceania, Australia	27	CF022383	Europe, Hungary
3	CF000056	Asia, Guizhou, China	28	CF022384	Europe, Italy
4	CF000108	Oceania, New Zealand	29	CF022385	Europe, Italy
5	CF000807	Europe, Denmark	30	CF022386	Europe, Romania
6	CF001320	Europe, Netherlands	31	CF022387	Europe, Lithuania
7	CF002737	North America, United States	32	CF022388	Europe, Lithuania
8	CF005832	Europe, Netherlands	33	CF022389	Europe, Lithuania
9	CF005835	Oceania, New Zealand	34	CF022390	North America, United States
10	CF005838	Oceania, New Zealand	35	CF022394	Europe, Italy
11	CF005842	Oceania, New Zealand	36	CF022398	Europe, France
12	CF005851	Oceania, New Zealand	37	CF022399	Europe, Greece
13	CF006886	Asia, Yunnan, China	38	CF022405	North America, United States
14	CF006887	Asia, Qinghai, China	39	CF022408	Europe, Italy
15	CF006900	Asia, Xinjiang, China	40	CF022409	Europe, France
16	CF008053	Oceania, Australia	41	CF022410	Europe, Greece
17	CF022345	Europe, Denmark	42	CF022412	Oceania, Australia
18	CF022347	Europe, Denmark	43	CF022413	North America, Canada
19	CF022350	Asia, Chongqing, China	44	CF022417	Asia, Beijing, China
20	CF022351	Asia, Sichuan, China	45	CF022418	Asia, Beijing, China
21	CF022365	Asia, Xinjiang, China	46	CF022419	Asia, Gansu, China
22	CF022367	Asia, Xinjiang, China	47	CF022428	Europe, Russia
23	CF022368	Europe, Czech Republic	48	CF022441	Asia, Beijing, China
24	CF022374	Europe, Czech Republic	49	CF022444	South America, Argentina
25	CF022379	Europe, United Kingdom	50	CF022448	North America, United States
**No.**	**Genotypes**	**Sampling Site**	**No.**	**Genotypes**	**Sampling Site**
51	CF022461	Europe, Russia	78	CF032120	Asia, Tajikistan
52	CF022480	Europe, Ukraine	79	CF032123	Asia, Tajikistan
53	CF022489	Europe, Russia	80	CF032126	Asia, Tajikistan
54	CF022510	Europe, Sweden	81	CF032128	Europe, Jexloval
55	CF022513	South America, Ulayao	82	CF032132	Asia, Kyrgyzstan
56	CF022514	South America, Brazil	83	CF032133	Asia, Azerbaijan
57	CF022519	Europe, Russia	84	CF032134	Asia, Armenia
58	CF022520	Europe, Russia	85	CF032143	North America, United States
59	CF022526	Europe, Russia	86	CF032144	South America, Brazil
60	CF022531	Europe, Russia	87	CF032145	South America, Peru
61	CF022533	Europe, Poland	88	CF032147	Europe, France
62	CF022551	Europe, Georgia	89	CF032149	Asia, Armenia
63	CF022559	Europe, United Kingdom	90	CF032150	Asia, Armenia
64	CF022562	Europe, Russia	91	CF032151	Europe, Georgia
65	CF022563	Europe, Russia	92	CF032154	Europe, Georgia
66	CF022565	Europe, Hungary	93	CF032156	Asia, Azerbaijan
67	CF022572	Europe, Latvia	94	CF032168	Asia, Kazakhstan
68	CF022573	Europe, Estonia	95	CF032171	Asia, Ukraine
69	CF022579	Europe, Spain	96	CF032172	Europe, Norway
70	CF022580	Europe, Spain	97	CF032173	Europe, Norway
71	CF022582	Asia, Kazakhstan	98	CF032174	Asia, Kyrgyzstan
72	CF022592	Europe, Russia	99	CF032176	Asia, Uzbekistan
73	CF025845	Asia, Sichuan, China	100	CF032181	Europe, United Kingdom
74	CF025851	Asia, Jilin, China	101	CF032187	Europe, Portugal
75	CF031027	Asia, China	102	CF032188	Europe, Portugal
76	CF031030	Asia, Jilin, China	103	CF032189	Europe, Portugal
77	CF031032	Europe, Netherlands	104	CF032193	Asia, Azerbaijan
**No.**	**Genotypes**	**Sampling Site**	**No.**	**Genotypes**	**Sampling Site**
105	CF032196	South America, Peru	132	CF040846	Europe, Sweden
106	CF032200	Europe, Serbia	133	CF040855	Asia, Hubei, China
107	CF032202	Europe, Russia	134	CF046128	Oceania, Australia
108	CF032204	Europe, Russia	135	CF046321	Asia, Jilin, China
109	CF032206	Europe, Kazakhstan	136	CF048084	Asia, Jilin, China
110	CF032207	Europe, Kyrgyzstan	137	CF048223	South America, Argentina
111	CF032209	Europe, Spain	138	CF048240	Asia, Xinjiang, China
112	CF032210	Europe, Latvia	139	CF048260	Asia, Nanjing, China
113	CF032211	Europe, Latvia	140	CF048271	Kunming, China, Asia
114	CF032213	Europe, Smolensk	141	CF049863	Europe, Russia
115	CF032216	Europe, Russia	142	CF049908	Europe, Sweden
116	CF032218	North America, Canada	143	CF049972	Europe, Russia
117	CF032222	Asia, Gansu, China	144	CF049984	Europe, Russia
118	CF032226	Asia, Anhui, China	145	CF050515	Asia, Shandong, China
119	CF032228	Europe, Germany	146	CF051265	Europe, Romania
120	CF032230	Europe, Germany	147	CF051266	Europe, Belgium
121	CF032232	Europe, Germany	148	CF051267	Europe, Belgium
122	CF037467	Asia, Heilongjiang, China	149	CF051268	Europe, Belgium
123	CF038677	Asia, Xinjiang, China	150	CF051269	Europe, Greece
124	CF038686	Asia, Xinjiang, China	151	EZWC025	Asia, Heilongjiang, China
125	CF038724	Asia, Xinjiang, China	152	EZWC065	Asia, Heilongjiang, China
126	CF038444	Asia, Liaoning, China	153	EZWC066	Asia, Heilongjiang, China
127	CF040329	Asia, Sichuan, China	154	EZWC067	Asia, Heilongjiang, China
128	CF040330	Asia, Sichuan, China	155	EZWC068	Asia, Heilongjiang, China
129	CF040839	Europe, Ukraine	156	HB2017018	Asia, Heilongjiang, China
130	CF040841	Europe, Estonia	157	HB2017019	Asia, Hubei, China
131	CF040842	Europe, Poland	158	HB2017020	Nanyang, China, Asia
**No.**	**Genotypes**	**Sampling Site**	**No.**	**Genotypes**	**Sampling Site**
159	HB2017036	Asia, Hubei, China	167	JL17-086	Asia, Heilongjiang, China
160	HB2017043	Asia, Hubei, China	168	JL17-087	Asia, China
161	HB2017063	Asia, Nanyang, China	169	JL17-088	Asia, China
162	HB2018022	Asia, Nanyang, China	170	JL18-076	Asia, China
163	HB2018028	Asia, Zhumadian, China	171	JL19-028	Asia, China
164	HB2018030	Asia, Nanyang, China	172	XJ2016-93	Asia, Xinjiang, China
165	HLJ-2017014	Asia, Nanyang, China	173	XJ2016-99	Asia, Xinjiang, China
166	HLJ-2017015	Asia, Heilongjiang, China	174	XJ2018-39	Asia, Xinjiang, China

## Data Availability

The data presented in this study are available on special request from corresponding author. The datasets generated and analyzed during the current study are available at NCBI under Submission ID: SUB16212315 and Bio Project ID: PRJNA1470920.

## Supplementary Materials

The following supporting information can be downloaded at https://www.mdpi.com/article/10.3390/ijms27114882/s1.
